# Supplementary material for: Prairie plants harbor distinct and beneficial root-endophytic bacterial communities
Source: PLoS One. 2020 Jun 23;15(6):e0234537. doi: 10.1371/journal.pone.0234537 (PMC7310688; doi:10.1371/journal.pone.0234537)
Supplement: S1 Table — (DOCX) [file pone.0234537.s007.docx]

**Supplemental Table S1A. ANOVA of Alpha Diversity Metric Root-Endophytic Bacterial Communities**. ANOVA with permutation for root-endophytic bacterial communities across all conditions (Autoclaved vs Non-Autoclaved) and Antibiotic Treatment.

| **Main Effects** | **Df** | **SumsOfSqs** | **MeanSqs** | **Iter** | **Pr(Prob)** |
| --- | --- | --- | --- | --- | --- |
| Condition (Autoclaved vs Non-Autoclaved) | 1 | 255553 | 255553 | 5000 | <2e-16 |
| Antibiotic Treatment | 1 | 352 | 352 | 51 | 1 |
| Residuals | 244 | 739824 | 3032 |  |  |

**Supplemental Table S1B. ANOVA of Total Dried Biomass**. ANOVA analysis for total dried biomass across all samples including the factors Condition (Autoclaved vs Non-Autoclaved), Antibiotic Treatment, Plant Host, Plot, and Soil History. Only interactions with significant effects are reported.

| Factor | Df | Sum of Squares | Mean Squares | F statistic | P value |  |
| --- | --- | --- | --- | --- | --- | --- |
| Condition (Autoclaved vs Non-Autoclaved) | 1 | 83.58 | 83.58 | 479.624 | <2e-16 | *** |
| Antibiotic Treatment | 1 | 0.07 | 0.07 | 0.38 | 0.5377 |  |
| Plant Host | 4 | 56.65 | 14.16 | 81.273 | <2e-16 | *** |
| Soil History | 4 | 2 | 0.5 | 2.871 | 2.27E-02 | * |
| Plot | 5 | 1.4 | 0.28 | 1.612 | 0.1552 |  |
| Condition: Plant Host | 4 | 83.63 | 20.91 | 119.974 | <2e-16 | *** |
