# Supplementary material for: Prairie plants harbor distinct and beneficial root-endophytic bacterial communities
Source: PLoS One. 2020 Jun 23;15(6):e0234537. doi: 10.1371/journal.pone.0234537 (PMC7310688; doi:10.1371/journal.pone.0234537)
Supplement: S2 Table — (DOCX) [file pone.0234537.s008.docx]

**Supplemental Table S2A. ADONIS of Endophytic Bacterial Communities**. ADONIS analysis for endophytic bacterial communities across all samples including the factors Condition (Autoclaved vs Non-Autoclaved), Antibiotic Treatment, Plant Host, Plot, and Soil History.

| **Main Effects** | **Df** | **SumsOfSqs** | **MeanSqs** | **F.Model** | **R2** | **Pr(>F)** |
| --- | --- | --- | --- | --- | --- | --- |
| Condition (Autoclaved vs Non-Autoclaved) | 1 | 5.932 | 5.9321 | 22.461 | 0.07836 | 0.001 |
| Antibiotic Treatment | 1 | 0.409 | 0.4093 | 1.5499 | 0.00541 | 0.026 |
| Plant Host | 4 | 5.562 | 1.3904 | 5.2645 | 0.07347 | 0.001 |
| Soil History | 4 | 1.467 | 0.3667 | 1.3884 | 0.01938 | 0.008 |
| Residuals | 236 | 62.329 | 0.2641 |  | 0.82338 |  |
| Total | 246 | 75.699 |  |  | 1 |  |

**Supplementary Table S2B. ADONIS of Endophytic Bacterial Communities of Plants Grown in Autoclaved Soils**. ADONIS analysis for endophytic bacterial communities across samples grown in autoclaved soils including the factors Antibiotic Treatment, Plant Host, and Soil History.

| **Main Effects** | **Df** | **SumsOfSqs** | **MeanSqs** | **F.Model** | **R2** | **Pr(>F)** |  |
| --- | --- | --- | --- | --- | --- | --- | --- |
| Plant Host | 4 | 0.9961 | 0.24903 | 3.5418 | 0.23678 | 0.001 | *** |
| Soil History | 4 | 0.4769 | 0.119225 | 1.6957 | 0.11336 | 0.004 | ** |
| Antibiotic Treatment | 1 | 0.2027 | 0.202686 | 2.8827 | 0.04818 | 0.003 | ** |
| Residuals | 36 | 2.5312 | 0.070312 |  | 0.60168 |  |  |
