# Supplementary material for: Prairie plants harbor distinct and beneficial root-endophytic bacterial communities
Source: PLoS One. 2020 Jun 23;15(6):e0234537. doi: 10.1371/journal.pone.0234537 (PMC7310688; doi:10.1371/journal.pone.0234537)
Supplement: S4 Table — (DOCX) [file pone.0234537.s010.docx]

**Supplemental Table S4. Tukey’s *post hoc* Total Dried Biomass for Soil History**. Tukey’s *post hoc* analysis for total dried biomass across all samples for the factor Soil History.

| **Comparison Soil History** | **Difference** | **Lower** | **Upper** | **P adjusted** |  |
| --- | --- | --- | --- | --- | --- |
| *C. canadensis – C. nutans* | -0.04309 | -0.18651 | 0.10032 | 0.923297 |  |
| *H. helianthoides - C. nutans* | 0.052663 | -0.0912 | 0.196526 | 0.853835 |  |
| *M. fistulosa – C. nutans* | 0.218191 | 0.074778 | 0.361604 | 0.000361 | *** |
| *R. pinnata – C. nutans* | 0.069687 | -0.07464 | 0.214011 | 0.676862 |  |
| *H. helianthoides – C. canadensis* | 0.095756 | -0.04766 | 0.239169 | 0.357821 |  |
| *M. fistulosa – C. canadensis* | 0.261284 | 0.118323 | 0.404246 | 8.2E-06 | *** |
| *R. pinnata – C. canadensis* | 0.11278 | -0.0311 | 0.256655 | 0.201977 |  |
| *M. fistulosa - H. helianthoides* | 0.165529 | 0.022115 | 0.308942 | 0.014452 | * |
| *R. pinnata - H. helianthoides* | 0.017024 | -0.1273 | 0.161348 | 0.997632 |  |
| *R. pinnata – M. fistulosa* | -0.1485 | -0.29238 | -0.00463 | 0.039222 | * |
