# Supplementary material for: Prairie plants harbor distinct and beneficial root-endophytic bacterial communities
Source: PLoS One. 2020 Jun 23;15(6):e0234537. doi: 10.1371/journal.pone.0234537 (PMC7310688; doi:10.1371/journal.pone.0234537)
Supplement: S6 Table — (DOCX) [file pone.0234537.s012.docx]

**Supplemental Table S6A. ANOVA of Total Dried Biomass**. ANOVA analysis for total dried biomass for *Monarda fistulosa* including the factors Condition (Autoclaved vs Non-Autoclaved). Only interactions with significant effects are reported.

| **Main Factors** | **Df** | **R Sum Sq** | **R Mean Sq** | **Iter** | **P-Value** |  |
| --- | --- | --- | --- | --- | --- | --- |
| Condition | 1 | 70.491 | 70.491 | 5000 | <2e-16 | *** |
| Residuals | 48 | 119.449 | 2.489 |  |  |  |

**Supplemental Table S6B. ANOVA of Total Dried Biomass**. ANOVA analysis for total dried biomass for *Heliopsis helianthoides* including the factors Condition (Autoclaved vs Non-Autoclaved). Only interactions with significant effects are reported.

| **Main Factors** | **Df** | **R Sum Sq** | **R Mean Sq** | **Iter** | **P-Value** |  |
| --- | --- | --- | --- | --- | --- | --- |
| Condition | 1 | 86.593 | 86.593 | 5000 | <2e-16 | *** |
| Residuals | 53 | 72.183 | 1.362 |  |  |  |

**Supplemental Table S6C. ANOVA of Total Dried Biomass**. ANOVA analysis for total dried biomass for *Ratibida pinnata* including the factors Condition (Autoclaved vs Non-Autoclaved). Only interactions with significant effects are reported.

| **Main Factors** | **Df** | **R Sum Sq** | **R Mean Sq** | **Iter** | **P-Value** |  |
| --- | --- | --- | --- | --- | --- | --- |
| Condition | 1 | 73.292 | 73.292 | 5000 | <2e-16 | *** |
| Residuals | 49 | 25.66 | 0.524 |  |  |  |
