# Supplementary material for: Prairie plants harbor distinct and beneficial root-endophytic bacterial communities
Source: PLoS One. 2020 Jun 23;15(6):e0234537. doi: 10.1371/journal.pone.0234537 (PMC7310688; doi:10.1371/journal.pone.0234537)
Supplement: S7 Table — (DOCX) [file pone.0234537.s013.docx]

**Supplementary Table S7A. ANOVA Using Permutation Tests of Total Dried Biomass**. ANOVA using permutation tests analysis for total dried biomass across all plant hosts including the factors Soil Type (Conspecific or Heterospecific).

| **Factor** | **Df** | **R Sum Sq** | **R Mean Sq** | **Iter** | **P-Value** |  |
| --- | --- | --- | --- | --- | --- | --- |
| Soil Type | 1 | 0.151 | 0.1506 | 51 | 1 |  |
| Plant Host | 4 | 121.424 | 30.3559 | 5000 | <2e-16 | *** |
| Soil Type x Plant Host | 4 | 17.114 | 4.2784 | 5000 | 0.0036 | ** |
| Residuals | 189 | 245.75 | 1.3003 |  |  |  |

**Supplementary Table S7B. ANOVA Using Permutation Tests of Total Dried Biomass**. ANOVA using permutation tests analysis for total dried biomass for *M. fistulosa* which were significant for factor Soil Type (Conspecific or Heterospecific).

| **Factor** | **Df** | **R Sum Sq** | **R Mean Sq** | **Iter** | **P-Value** |  |
| --- | --- | --- | --- | --- | --- | --- |
| Soil Type | 1 | 8.588 | 8.588 | 2006 | 0.04786 | * |
| Residuals | 37 | 74.532 | 2.0144 |  |  |  |

**Supplementary Table S7C. ANOVA Using Permutation Tests of Total Dried Biomass**. ANOVA using permutation tests analysis for total dried biomass for *C. canadensis* which were significant for factor Soil Type (Conspecific or Heterospecific).

| **Factor** | **Df** | **R Sum Sq** | **R Mean Sq** | **Iter** | **P-Value** |  |
| --- | --- | --- | --- | --- | --- | --- |
| Soil Type | 1 | 6.374 | 6.3739 | 5000 | 0.016 | * |
| Residuals | 38 | 41.713 | 1.0977 |  |  |  |
