# Supplementary material for: Prairie plants harbor distinct and beneficial root-endophytic bacterial communities
Source: PLoS One. 2020 Jun 23;15(6):e0234537. doi: 10.1371/journal.pone.0234537 (PMC7310688; doi:10.1371/journal.pone.0234537)
Supplement: Attachment — Submitted filename: 200223_response_to_reviewers_v5_BA.docx [file pone.0234537.s015.docx]

*Currently, early in the Results (line 356), the authors state “even though we did not demonstrate significant plant soil-feedback” but no analyses or information are presented. Readers have to wait until lines 455-465 to understand the details of the feedback results. The authors should think about the order in which they present material. If feedback is central to the paper, I would present this information early in the Results.*

Thank you for bringing this to our attention. We have removed the comment about plant soil-feedbacks here since those specific results are not discussed until later (following a parallel structure based on the 4 questions we posed at the end of the introduction).

The new line is below:

To determine if specific soil histories affected plant performance, we measured the dried biomass of all plants grown in each of the field-soil inocula. (Lines 356-357)

*I also think lines 577 – 578 will be confusing to many (i.e., the distinction between the two ways of talking about feedback).*

Thank you for that insightful comment. We have added more context and the new line is below:

In contrast to other studies, we did not detect a significant main effect plant-soil feedback (feedback between all plant hosts) within our experiment (S5 Fig); however, we did detect pair-wise plant-soil feedbacks between pairs of plant hosts (Fig 6). (Lines 578 - 580)


*36 – 37 – suggest rewrite “; in contrast to the degree to which ……”*

Following your suggestion we have re-written the sentence:

“Most studies focus on the microbial community in the soil rhizosphere; therefore, the degree to which the bacterial community within plant roots (root-endophytic compartment) influences plant-microbe interactions remains relatively unknown.” (Lines 36 – 38)

*38 – I would have said “influences”*

We have changed the word to “influences”. Please see above, we have changed this as you suggested.

*71 – suggest rewrite “relationships between the plant and the microbiome”*

*73 – long sentence – perhaps delete phrase after comma*

To address both suggestions, we have rewritten this part as:

“Second, few studies perturb the root-endophytic bacterial community with mechanisms which drastically impact the composition. To understand the relationships between the plant and the microbiome (34-37), experiments should include treatments which affect both the microbiome and the plant. Instead, many focus on perturbations that directly affect plant community dynamics.” (Lines 70 – 74)

*78 – suggest rewrite “……….such as drought. This is important given that current predictions state that the intensity…………”*

We have updated the sentence to read:

“Many studies suggest that the root-endophytic bacterial community helps plants mitigate abiotic stress (32, 40-43) such as drought. This is important because the intensity and frequency of droughts are predicted to increase due to climate change (44, 45).” (Lines 76 – 79)

*81 – suggest rewrite “………soil microbial diversity (as expected with antibiotic use) leads to……*

*81 – suggest rewrite “………..performance, and even fewer”*

*To address both suggestions, we have re-written this part as:*

“Few studies have shown that a decrease in soil microbial diversity (as expected with antibiotic use) leads to decreases in plant performance, and even fewer have linked this to root-endophytic bacterial communities (2, 37).” (Lines 80 – 83)

*95 – I might delete “must” (I know this is a part of feedback, but sentence structure seems awkward with it present)*

We have removed the word “must”. The new sentence reads as:

“The term feedback within the scope of this study involves 2 steps: 1) the plant host perturbs the composition of the bacterial community, and 2) this differentiation affects the performance of the plant host (6).” (Lines 94 – 96)

*98 – add comma after herbivore*

We have included the comma:

“Plant-soil feedbacks can predict co-existence of plant species since feedbacks are plant host-specific and can either be negative or positive (22) depending on the balance of negative effects of soil-borne pathogens, herbivores, and parasites compared to positive effects of beneficial soil microbes (60).” (Lines 96 – 99)

*100 – add “plant” before the word “species”*

We have taken this suggestion:

“…thus limiting dominance and competition among plant species.” (Lines 101)

*109 – I haven’t read this book but 13% seems too high. Often one sees Samson and Knopf Bioscience 44:418-421 cited, with a reference to 1-4% prairies left. It is tricky – tables in this article show 1% prairie left in most states and more in a few states. These articles are also quite old – more prairie has been lost in the last 20 years. But alas, a really good reference doesn’t exist to my knowledge.*

Yes, we haven’t been able to find a more recent citation. Most papers including this one published in 2017 still references the Samson and Knopf paper: North American tallgrass prairies represent one of the most widely destroyed and degraded ecosystems on Earth, with >90% of original prairie lost, and in some regions such as Illinois, >99% (Samson and Knopf, [1994](https://sfamjournals.onlinelibrary.wiley.com/doi/full/10.1111/1462-2920.13785#emi13785-bib-0054); Samson and Knopf, [1996](https://sfamjournals.onlinelibrary.wiley.com/doi/full/10.1111/1462-2920.13785#emi13785-bib-0055)). (Barber et al 2017 Environmental Microbiology <https://doi.org/10.1111/1462-2920.13785>)

*129 – need spaces between 1 and invasive*

We have added the space.

“We chose 5 prairie species, 4 natives: *Monarda fistulosa* (Wild Bergamot), *Ratibida pinnata* (Grey-head coneflower), *Heliopsis helianthoides* (Smooth oxeye), *Conyza canadensis* (Horseweed); 1 invasive which was listed as a noxious weed in Missouri (USDA 2019): *Carduus nutans* (Musk Thistle).” (Lines 127 – 130)

*149 – suggest rewrite “(step 1 of the plant-feedback framework)”*

*150 - do you mean “sample closer to adult plants in field sites”??*

Yes, we do mean in field sites. We have included that in the sentence to make it clearer.

To address the last two comments we have re-written this sentence as:

“Step 1 of the plant-feedback framework is to create differentiated soil communities by either allowing plant hosts to grow in similar initial soil communities for a few months or sampling close to adult plants in field sites due to the short generation time and rapid community dynamics of microbial communities (54).” (Lines 149 – 152)

*160 – Is this right? I would have expected soils to be pooled across experimental plots for those collected under the same plant species?*

Yes, we did not pool the plots. We kept them separated and included that factor as a variable within our statistical model.

*201-2 - It is stated that plant performance was not affected when grown in field autoclaved or field soil, but I wasn’t sure whether this was a specific result of this study (if so, I’d expect a phrase like “See results” at the end) or whether this was a general statement (I’d expect to see a citation or at least xxx, unpublished data)*

Yes, you are correct. We have added the reference to our figure in the supplements that demonstrate this result:

“Antibiotics were chosen as a perturbation due to their ability to directly affect microbial communities by eliminating species from the communities without directly impacting plant growth (S1 Fig). Plant performance was not affected when grown in the presence or absence of antibiotics (S1B Fig).” (Lines 199 – 202)

*219 Typo – “experiment” not “experimental”*

Thank you for catching this typo. We have changed it to “experiment” instead of “experimented”. (Line 222)

*229 – suggest rewrite “above-ground plant parts”*

We have updated the sentence to reflect the suggested change:

“To calculate plant-soil feedback interaction coefficient ($I_{s})$, we used the dried biomass of both above-below ground plant parts and calculated the interaction using this equation (71):” (Lines 228 – 229)

*248 – not sure of the meaning of root position*

We did not standardize where we collected the root across all plants. We have re-worded the sentence to make this clear:

“Since we wanted to limit the amount of cross contamination, we did not standardize the exact location of the extracted root sample across all plants.” (Lines 247 – 249)

*250 insert “the” before “portion”*

We have included the word “the” where suggested. (Line 258)

*263 insert “the” before “microbial community”*

We have included the word “the” where suggested. (Line 271)

*306 perhaps insert “additionally” before “correlated” if the goal is to show this is an additional strength of the study*

Yes, thank you for the suggested change. The new sentence is:

“We additionally correlated the composition of the bacterial communities to plant performance to understand if differences in endophytic root bacterial compositions could explain differences in plant biomass.” (Lines 306 – 308)

*308 insert “the” before “linear”*

We have included the word “the” where suggested. (Line 318)

*335 suggest rewrite “was better explained (as indicated by the r2 value) by plant identity…………..*

We have changed the placement of the r^2^ value to the suggested placement. The new sentence is:

“In a subset of samples which were non-autoclaved and non-antibiotic treated, the compositional differences in root bacterial community was better explained (as indicated by the higher r^2^ value) by plant identity (ADONIS p < 0.001, r^2^=0.11, Fig 2A) than by soil history (ADONIS p < 0.001, r^2^=0.03, Fig 2B).” (Lines 333 – 336)

*357 there is a problem with the flow of this sentence*

Thank you for bringing this to our attention. The new sentence is found below:

“To determine if specific soil histories affected plant performance, we measured the dried biomass of all plants grown in each of the field-soil inocula.” (Lines 356 – 357)

*394 replace comma with semi colon (or more generally think about how to reduce the complexity and structure of this sentence)*

We reduced the complexity by creating two sentences. The new sentences are:

“We can attribute plant performance to the soil biotic component because we controlled for potential variation in abiotic properties introduced by the small volume of soil history. Additionally, we assessed seedling growth in the same plant-inoculum combinations but with autoclaved inoculum.” (Lines 391 – 394)

*407 – 410 I understand the meaning of the sentence but it is cumbersome to read.*

To make this sentence easier to read, we removed some of the stats which can be found on the figure and referred to the figure just once. The new sentences are:

“*C. nutans* (p < 0.013), *H. helianthodies* (p < 0.001), *M. fistulosa* (p < 0.01,), and *R. pinnata* (p < 0.0001), but not *C. canadensis* (p = 0.7), demonstrated a correlation between biomass and community similarity (Fig 4).” (Lines 406 – 408)

*445-446 Reword. The reference to “we perturb” makes it sound like a methods sentence but* clearly it is a results sentence

We have reworded this sentence:

“To understand the specificity of the root endophytic microbial community to the host, we measured the change in root endophytic microbial community composition after exposure to antibiotics (ADONIS p = 0.26, r^2^ = 0.005) and the autoclave treatment (ADONIS p < 0.001, r^2^ = 0.07).” (Lines 444 – 447)

*503 – It seems odd to emphasize the idea that individual plants within the same species are different before addressing the larger point that different host species structure the microbial community*

Thank you for bringing this to our attention. We have rephrased to highlight the larger point and end with the individual plants. The new sentences are:

“Within our study, we discovered that individuals within each plant host are more similar to each other than to different plant hosts (Fig 2A). Moreover, since we used natural variation within each plant host rather than artificially manipulating the plant genome and noticed large variation between individuals within each plant host indicating that root endophytic bacterial microbiome is not identical within all individuals of the same plant host (Fig 2A).” (Lines 499 – 504)

*559 - should say “would become dominant.” Also, I think it is too much of a stretch to infer from a single lab study with autoclaving whether particular species would be more common after a fire. Many factors affect post-fire response.*

Thank you for bringing this to our attention. We have changed the word “dominate” to “dominant”. Additionally, we have added the following sentences to highlight your point that more studies need to be conducted to understand the impact rather than using our study as the rule of thumb:

“This result suggests that certain plant species would become dominant while others would become rare within the population after a prescribed fire.” (Lines 559 – 561)

“Additional studies are needed to understand the impact of current prairie restoration practices on root-endophytic bacterial communities and its impact on prairie community composition.” (Lines 564 – 566)
